# A Fusion Algorithm for Estimating Time-Independent/-Dependent Parameters and States

**DOI:** 10.3390/s21124068

**Published:** 2021-06-12

**Authors:** Zheshuo Zhang, Jie Zhang, Jiawen Dai, Bangji Zhang, Hengmin Qi

**Affiliations:** State Key Laboratory of Advanced Design and Manufacturing for Vehicle Body, College of Mechanical and Vehicle Engineering, Hunan University, Changsha 410082, China; zszhang@hnu.edu.cn (Z.Z.); jiawendai@hnu.edu.cn (J.D.); bangjizhang@hnu.edu.cn (B.Z.); qihm@hnu.edu.cn (H.Q.)

**Keywords:** vehicle dynamics, real-time parameter estimation, dual unscented Kalman filter, modal analysis, vehicle parameter identification

## Abstract

Vehicle parameters are essential for dynamic analysis and control systems. One problem of the current estimation algorithm for vehicles’ parameters is that: real-time estimation methods only identify parts of vehicle parameters, whereas other parameters such as suspension damping coefficients and suspension and tire stiffnesses are assumed to be known in advance by means of an inertial parameter measurement device (IPMD). In this study, a fusion algorithm is proposed for identifying comprehensive vehicle parameters without the help of an IPMD, and vehicle parameters are divided into time-independent parameters (TIPs) and time-dependent parameters (TDPs) based on whether they change over time. TIPs are identified by a hybrid-mass state-variable (HMSV). A dual unscented Kalman filter (DUKF) is applied to update both TDPs and online states. The experiment is conducted on a real two-axle vehicle and the test data are used to estimate both TIPs and TDPs to validate the accuracy of the proposed algorithm. Numerical simulations are performed to further investigate the algorithm’s performance in terms of sprung mass variation, model error because of linearization and various road conditions. The results from both the experiment and simulation show that the proposed algorithm can estimate TIPs as well as update TDPs and online states with high accuracy and quick convergence, and no requirement of road information.

## 1. Introduction

Parameters are very important for dynamic analysis and control systems [1], as the accuracy of parameters directly determines the precision of the analysis results [2,3] and the performance of control systems [4,5]. Parameters can be divided into time-independent parameters (TIPs) and time-dependent parameters (TDPs) based on whether they change over time. TIPs are fixed values specified by a vehicle manufacturer, including inertial parameters of empty vehicles, suspension damping coefficients and suspension and tire stiffnesses. By contrast, TDPs can vary during a trip, including real-time sprung mass, center of gravity (CG) and moment of inertia (MI).

An inertial parameter measurement device (IPMD) is a highly specialized test device for the measurement of vehicle TIPs, and thus is expensive [6]. Generally, TIPs can be supplied by the vehicle manufacturer. But for a situation where TIPs are not supplied, an estimation method of TIPs without limitation of the IPMD would be practical and easy to use. Zheng et al. [7] suggest an estimation process of parameters based on the free-decay responses of the vehicle; the test can be performed on anywhere existing a short stage. If the modal parameters are obtained, then the gross mass, suspension damping coefficients and suspension and tire stiffnesses can be determined by a fitting process. The modal parameter identification methods can be divided into frequency-domain methods and time-domain methods. As the modal frequencies of the vehicle body are typically in 1–3 Hz, which is a small range, frequency domain methods are limited, whereas time domain methods are more appropriate to identify the modal parameters [8].

Relatively, TDPs’ estimation is more challenging as an online process is necessary. For example, although the mass and CG can be calculated using a static weighing scale, the online information of the vehicle mass is hard to know due to pick-ups or drop-offs during a trip and dynamic behaviors in motion. Rozyn and Zhang [9] suggested an updated TDP method with the sprung mass response by extracting equivalent free-decay responses, which essentially is still based on the identification of modal parameters. The limitation of this method is that extraction of free-decay responses will affect real-time performance.

Real-time estimation methods for some of the vehicle parameters have been suggested by numerous studies. Recursive least square (RLS) is a popular one for vehicle mass, which utilizes maximum likelihood theory [10,11,12,13]. A Kalman filter (KF) is also commonly used [14,15,16,17,18] as a special case of the recursive Bayesian estimation algorithm with assumptions of linear state-space representation and Gaussian probability distribution. However, both RLS and the KF are limited for linear systems, and hence, KF variants, such as extended KF [19,20,21,22,23] or unscented KF [24,25,26], are more suitable for nonlinear estimation. The extended KF usually provides “first-order” approximations to the optimal terms. While “second-order” versions of the extended KF exist, their increased implementation and computational complexity tend to prohibit their use. The unscented transform (UT) is a method for calculating the statistics of a random variable that undergoes a nonlinear transformation. In addition, a two-layer algorithm is also suggested: one layer is for vehicle parameters, while the other one is for states [24,27]. Therefore, a dual unscented KF method would be very useful to deal with both parameters and states for nonlinear systems.

The vehicle parameter estimation methodologies can also be classified based on the vehicle models used: longitudinal, yaw, roll and bounce. The most prevalent methods in preview studies are based on the longitudinal one [10,11,12,13,28], which appears simple though is practically limited by access to input forces, such as changing vehicle gravity, aerodynamic drag and road friction. In addition, only vehicle mass but neither CG nor MI can be estimated by the longitudinal model method. The yaw and roll models are also popular [19,24,29,30] but their accuracy is significantly dependent on the accuracy of the tire model, which is highly variable according to tire brand, wear and inflation pressures. Theoretically, both the vehicle parameters and suspension states can be accurately estimated through a vertical model [9], but few studies for vehicle parameter estimation are found using this kind of model. Boada et al. [29] estimated mass through a vertical model, but they ignored the variation of CG and MI when load mass changed. Zhou et al. [30] suggested a very interesting mass estimation method based on vertical suspension deflection recognition with cameras, but nevertheless averaged a 42.5% relative error, likely caused by non-contact measurement.

Taking into consideration all the previous ideas, the primary objective of this study is to propose a comprehensive algorithm estimating TIPs as well as updating TDPs and online states with high accuracy and quick convergence. The contributions that clearly distinguish our endeavor from the aforementioned literature are that vehicle parameters are properly divided into TIPs and TDPs according to the requirement for updates, and consequently, different methods are formulated. TIPs can be determined offline based on the identification of modal parameters, while TDPs can be estimated online based on a real-time dual unscented Kalman filter (DUKF).

The outline of the paper is as follows. Section 2 formulates the vehicle model in vertical, pitch and roll dynamics. Section 3 describes the hybrid-mass state-variable (HMSV) method for TIPs’ estimation based on the identification of modal parameters. The structure of the DUKF for the online identification of TDPs and states is presented in Section 4. In Section 5, the proposed algorithm is verified by experimental data. Both a simulation and parameter study are shown in Section 6 to prove the algorithm’s performance in terms of sprung mass variation, model error because of linearization, and various road conditions. Our conclusion is summarized in Section 7.

## 2. Vehicle Model in Vertical and Pitch Dynamics

**Figure 1:**
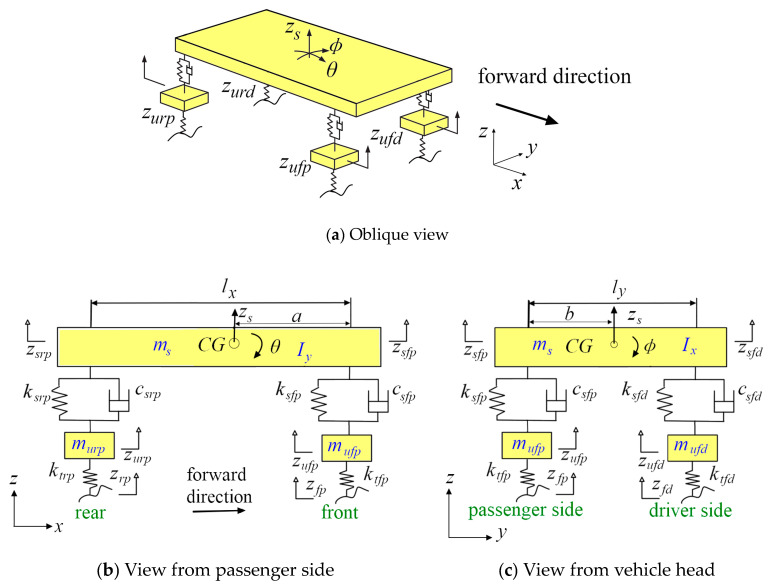
Multi-body dynamics model of full vehicle.

A full vehicle model comprising two axles is illustrated in Figure 1. The model consists of seven DOF (degrees of freedom), corresponding to the vertical motion of sprung mass $z_{s}^{}$, pitch motion of sprung mass θ, roll motion of sprung mass ϕ and vertical motions of four unsprung masses: $z_{ufd}$, $z_{ufp}$, $z_{urd}$ and $z_{urp}$. The subscript s stands for sprung mass, while u is for unsprung mass, f is for front axle, r is for real axle, d is for driver side and p is for passenger side. $z_{i}$ (i=fp,fd,rp,rd) stands for the road profile input of each wheel. A detailed description of the vehicle parameters is given in Table A1 and Table A2 in Appendix A.

The seven DOF vehicle model can be described by the following dynamic equation

(1) $$M\ddot{X}+C\dot{X}+KX=F_{ext}+F_{non}$$

where $\ddot{X}$, $\dot{X}$ and X are acceleration, velocity and displacement vectors, respectively; M, C and K are mass, damping and stiffness matrices, respectively; and vectors $F_{ext}$ and $F_{non}$ stand for the external excitation and suspension nonlinear parts of the vehicle system, respectively. These matrices and vectors are given by:

(2) $$X={\left[z_{s}\;\theta \;\phi \;z_{ufd}\;z_{ufp}\;z_{urd}\;z_{urp}\right]}^{T},$$

(3) $$M=\mathrm{diag}\left(\begin{array}{lllllll} m_{s} & I_{y} & I_{x} & m_{ufd} & m_{ufp} & m_{urd} & m_{urp} \end{array}\right)$$

(4) $$C=\left[\begin{array}{l} LC_{s}L^{T}-LC_{s}\\ -C_{s}L^{T}\;\;\;C_{s} \end{array}\right],$$

(5) $$K=\left[\begin{array}{l} LK_{s}L^{T}-LK_{s}\\ -K_{s}L^{T}\;\;K_{s}+K_{t} \end{array}\right],$$

(6) $$F={\left[\begin{array}{lllllll} 0 & 0 & 0 & k_{tfd}z_{fd} & k_{tfp}z_{fp} & k_{trd}z_{rd} & k_{trp}z_{rp} \end{array}\right]}^{T}$$

where the superscript “*T*” denotes the transposition of a vector or matrix, “diag” stands for diagonal matrix and the matrices $C_{s}$, $K_{s}$, L and $K_{t}$ are expressed as follows, respectively

(7) $$C_{s}=\mathrm{diag}\left(c_{sfd},\;c_{sfp},\;c_{srd},\;c_{srp}\right),$$

(8) $$K_{s}=\mathrm{diag}\left(k_{sfd},\;k_{sfp},\;k_{srd},\;k_{srp}\right),$$

(9) $$K_{t}=\mathrm{diag}\left(k_{tfd},\;k_{tfp},\;k_{trd},\;k_{trp}\right),$$

(10) $$L=\left[\begin{array}{cccc} 1 & 1 & 1 & 1\\ -a & -a & \left(l_{y}-a\right) & \left(l_{y}-a\right)\\ -\left(l_{x}-b\right) & b & -\left(l_{x}-b\right) & b \end{array}\right]$$

## 3. HMSV for Time-Independent Parameters

In this section, the time-independent parameters (TIPs), listed in Table A1 in Appendix A, are determined by the hybrid-mass state-variable (HMSV) method.

### 3.1. State-Variable Method

The basic concept of HMSV is identifying parameters through modal parameters, and thus the state-variable (SV) method is embedded to determine the modal parameters, firstly.

Introducing state vector $Y={\left[X\dot{X}\right]}^{T}$, Equation (1) can be converted to the state-space form:

(11) $$\dot{Y}=AY+B\left(F_{ext}+F_{non}\right),$$

where $F_{ext}$ is the road input, $F_{non}$ is the suspension nonlinear force, and matrices A and B are

(12) $$A=\left[\begin{array}{cc} 0_{7\times 7} & I_{7\times 7}\\ -M^{-1}K & -M^{-1}C \end{array}\right]$$

(13) $$B=\left[\begin{array}{c} 0_{7\times 7}\\ M^{-1} \end{array}\right]$$

During the free decay response, the road input is $F_{ext}=0$. In addition, the vehicle suspension usually expresses a strong linear character around the working point, while the nonlinear part generally can be ignored in the vehicle models [31], therefore, assuming $F_{non}=0$. Consequently, the state-space equation can be described in the discrete time domain:

(14) $$Y\left(k\right)=A_{1}Y\left(k-1\right),$$

where

(15) $$Y\left(k-1\right)={\left[X^{T}\left(k-1\right)X^{T}\left(k\right)\right]}^{T},$$

(16) $$Y\left(k\right)={\left[X^{T}\left(k\right)X^{T}\left(k+1\right)\right]}^{T},$$

(17) $$A_{1}=e^{dtA}.$$

dt is the sampling interval. A finite difference equation is constructed with the transition matrix $A_{1}$ as:

(18) $$\Pi =A_{1}\Gamma +R$$

where R represents the measure error matrix, and the state matrices Π and Γ are defined as

(19) Γ=[Y(l), Y(2),⋯, Y(N)]

(20) Π=[Y(2), Y(3),⋯, Y(N+1)].

*N* is the column number of Γ. Based on the least squares method, the transition matrix $A_{1}$ can be determined by

(21) $$A_{1}=\left(\Pi \Gamma^{T}\right){\left(\Gamma \Gamma^{T}\right)}^{-1}$$

Solving the eigenvalue problem of the transition matrix $A_{1}$ gives the eigenvalues $\lambda^{A1}{}_{i}$ and eigenvectors $U^{A1}{}_{i}$ (i=1,2,⋯,7). Based on Equation (17), and the eigenvalues $\lambda^{A}{}_{i}$ and eigenvectors $U^{A}{}_{i}$ of matrix A can be calculated as

(22) $$\lambda^{A}{}_{i}=\frac{\ln\left(\lambda^{A1}{}_{i}\right)}{dt},\;(i=1,2,\cdots ,7)$$

(23) $$U^{A}{}_{i}=U^{A1}{}_{i},\;(i=1,2,\cdots ,7)$$

The matrix A can be calculated by

(24) $$A=\left[\begin{array}{cc} U^{A} & U^{A}{}^{*}\\ U^{A}\Lambda & U^{A}{}^{*}\Lambda^{*} \end{array}\right]\left[\begin{array}{ll} \Lambda & \\ & \Lambda^{*} \end{array}\right]{\left[\begin{array}{cc} U^{A} & U^{A}{}^{*}\\ U^{A}\Lambda & U^{A}{}^{*}\Lambda^{*} \end{array}\right]}^{-1}$$

where $\Lambda^{*}$ and $U^{A}{}^{*}$ are, respectively, the conjugate matrices of Λ and $U^{A}$:

(25) $$\Lambda =\mathrm{diag}\left[\begin{array}{llll} \lambda^{A}{}_{1} & \lambda^{A}{}_{2} & \cdots & \lambda^{A}{}_{7} \end{array}\right]$$

(26) $$U^{A}=\left[\begin{array}{llll} U^{A}{}_{1} & U^{A}{}_{2} & \cdots & U^{A}{}_{7} \end{array}\right]$$

### 3.2. Determining Parameters with HMSV Method

The state matrix A of the original vehicle system can be solved by Equation (24) but it is still not possible to determinate the mass matrix M, the damping coefficient matrix C or the stiffness matrix K of the vehicle by directly solving Equation (12) alone, as the number of equations is fewer than the number of unknowns. Therefore, a hybrid-mass state-variable (HMSV) method is suggested by putting additional mass into the original vehicle system [7], forming a new mass matrix $M_{new}$. Then, the new state matrix $A_{new}$ can be represented as

(27) $$A_{new}=\left[\begin{array}{cc} 0_{7\times 7} & I_{7\times 7}\\ -M_{new}{}^{-1}K & -M_{new}{}^{-1}C \end{array}\right]$$

Both A in Equation (12) and $A_{new}$ in Equation (27) can be determined by the SV method. Set the submatrices $A^{sub}$ and $A_{new}^{sub}$ of A and $A_{new}$, respectively, are

(28) $$A^{sub}=-M^{-1}K$$

(29) $$A_{new}^{sub}=-M_{new}{}^{-1}K$$

Combining Equation (28) and Equation (29), there is

(30) $$M=\left(M_{new}-M\right)A_{new}^{sub}{\left(A^{sub}-A_{new}^{sub}\right)}^{-1}.$$

As the additional mass and position can be measured, $\left(M_{new}-M\right)$ is easily calculated. Therefore, the mass matrix M can be derived by Equation (30). Substituting M into Equation (12), the damping coefficient matrix C and the stiffness matrix K of the vehicle can be determined. Subsequently, all fixed parameters of Table A1 in Appendix A can be obtained, including the inertial parameters of the empty vehicle, suspension damping coefficients, suspension stiffnesses and tire stiffnesses.

## 4. DUKF for Time-Dependent Parameters and States

In this section, the estimation process for TDPs and states using a DUKF method are formulated in detail. For a changing loading condition, all TDPs—$m_{s}$, Δm, a, b, $I_{x}$ and $I_{y}$ (listed in Table A2 in Appendix A)—are time-dependent and need to be estimated in real-time. Among them, $m_{s}$, a and b are firstly estimated by the DUKF method, and then online MIs and Δm can be calculated by the relationship between these vehicle inertial parameters, so as to be less complex than all TDPs estimated together.

### 4.1. Relationship between Time-Dependent Parameters

**Figure 2:**
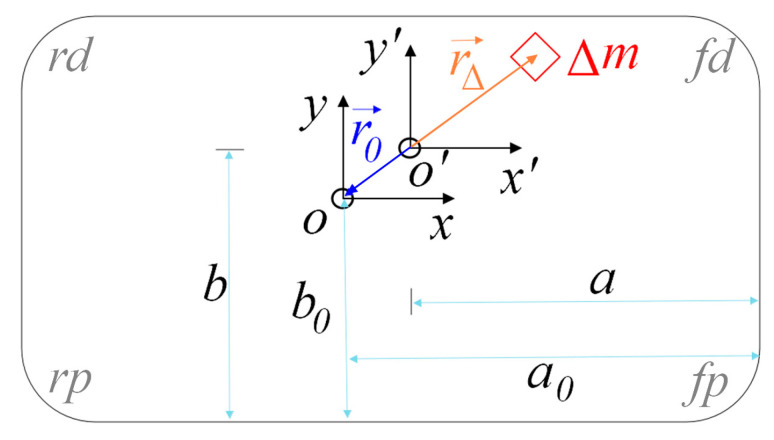
Relationship between vehicle inertial parameters.

The additional mass Δm is assumed as a rigid body, and it results in a new total sprung mass $m_{s}=m_{0}+\Delta m$, as well as the new MIs ($I_{x}$ and $I_{y}$) and the new CG displacements (a and b). The coordinate frame oxy is at the original CG of the empty vehicle’s sprung mass, while the coordinate frame $o^{\prime }x^{\prime }y^{\prime }$ is at the new CG, as illustrated in Figure 2.

As shown in Figure 2, in the coordinate frame, $o^{\prime }x^{\prime }y^{\prime }$, ${\vec{r}}_{0}$ and ${\vec{r}}_{\Delta }$ are vectors from $o^{\prime }$ to *o* and added mass, respectively. As $o^{\prime }$ is the new mass center combining $m_{0}$ and Δm, there will be ${\vec{r}}_{0}\cdot m_{0}+{\vec{r}}_{\Delta }\cdot \Delta m=0$, and thus ${\vec{r}}_{0}=\left(a-a_{0}\right)\cdot {\vec{e}}_{x^{\prime }}+\left(b-b_{0}\right)\cdot {\vec{e}}_{y^{\prime }}$, where ${\vec{e}}_{x^{\prime }}$ and ${\vec{e}}_{y^{\prime }}$ are unit vectors in the $x^{\prime }$- and $y^{\prime }$- directions, respectively. The added mass position can be expressed as

(31) $${\vec{r}}_{\Delta }=\frac{m_{0}}{\Delta m}\cdot \left[\left(a_{0}-a\right)\cdot {\vec{e}}_{x^{\prime }}+\left(b_{0}-b\right)\cdot {\vec{e}}_{y^{\prime }}\right]$$

Using the parallel-axis theorem, the new MIs can be calculated as

(32) $$\left\{ \begin{array}{l} I_{x}=\left[I_{x0}+m_{0}\cdot {\left(b-b_{0}\right)}^{2}\right]+\Delta m\cdot {\left({\vec{r}}_{\Delta }\cdot {\vec{e}}_{y^{\prime }}\right)}^{2}\\ I_{y}=\left[I_{y0}+m_{0}\cdot {\left(a-a_{0}\right)}^{2}\right]+\Delta m\cdot {\left({\vec{r}}_{\Delta }\cdot {\vec{e}}_{x^{\prime }}\right)}^{2} \end{array}\right.$$

Substituting Equation (31) and $m_{s}=m_{0}+\Delta m$ into Equation (32), the new MIs can be expressed by estimated $m_{s}$, *a* and *b* as

(33) $$\left\{ \begin{array}{l} I_{x}=I_{x0}+\frac{m_{0}\cdot m_{s}}{m_{s}-m_{0}}\cdot {\left(b-b_{0}\right)}^{2}\\ I_{y}=I_{y0}+\frac{m_{0}\cdot m_{s}}{m_{s}-m_{0}}\cdot {\left(a-a_{0}\right)}^{2} \end{array}\right.$$

### 4.2. Structure of Dual Unscented Kalman Filter

The structure of the DUKF is detailed herein based on the system model formulated in Section 2 and the SIPs determined in Section 3. Whilst a dual Kalman filter and dual extended Kalman filter were proposed by Wan and Nelson [32,33], they were applied to estimate vehicle states and part of the parameters by [19,24,29,34]. The DUKF in this paper uses the unscented KF (UKF) to ensure accuracy for both the linear and nonlinear systems. The DUKF is formed by two filters: the states UKF and the SIPs UKF.

The system equation for the states UKF is:

(34) $$\left\{ \begin{array}{l} x_{s,k}=f\left(x_{s,k-1},x_{p,k-1}^{}\right)+w_{s,k}\\ y_{k}=h\left(x_{s,k},x_{p,k-1}^{}\right)+v_{k} \end{array}\right.$$

and the system equation for TDPs UKF is:

(35) $$\left\{ \begin{array}{l} x_{p,k}=x_{p,k-1}^{}+w_{p,k}\\ y_{k}=h\left(f\left(x_{s,k-1},x_{p,k}\right),x_{p,k}\right)+v_{k} \end{array}\right.$$

$w_{s}$ and $w_{p}$ are the process noise vector of states and TDPs, assumed to be zero mean Gaussian white noise with covariance $Q_{s}$ and $Q_{p}$, respectively. $Q_{s}=\lambda_{Q}I$, and $\lambda_{Q}$ is set to a large enough value, which is equal to 1000 in the later experiment and simulation; $Q_{p}=\left[\sigma_{TDPs}^{2}\right]$, where $\sigma_{TDPs}^{}$ is about 1% of the corresponding initial value of TDPs [29]. The state vector for the states UKF is set as:

(36) $$x_{s}={\left[\;{\dot{z}}_{s},\;\dot{\theta },\;\dot{\phi },\;{\dot{z}}_{ufd},\;{\dot{z}}_{ufp},\;{\dot{z}}_{urd},\;{\dot{z}}_{urp},\;\Delta_{sfd},\;\Delta_{sfp},\;\Delta_{srd},\;\Delta_{srp},\;\Delta_{tfd},\;\Delta_{tfp},\;\Delta_{trd},\;\Delta_{trp}\right]}^{T}.$$

$\Delta_{si}=z_{si}-z_{ui}$ and $\Delta_{ti}=z_{ui}-z_{i}$ (i=fp,fd,rp,rd) stand for the dynamic deflections of suspensions and tires, respectively. The sprung vertical displacement $z_{si}$(i=fp,fd,rp,rd) at the top of each suspension can be expressed as:

(37) $$\left[\begin{array}{l} z_{sfp}\\ z_{sfd}\\ z_{srp}\\ z_{srd} \end{array}\right]=\left[\begin{array}{l} z_{s}-\theta a+\phi b\\ z_{s}-\theta a-\phi (l_{x}-b)\\ z_{s}+\theta \left(l_{y}-a\right)+\phi b\\ z_{s}+\theta \left(l_{y}-a\right)-\phi (l_{x}-b) \end{array}\right].$$

In this way, the road profile inputs $z_{i}$(i=fp,fd,rp,rd) are implicitly expressed as states in $\Delta_{ti}$(i=fp,fd,rp,rd), which can be estimated rather than required as an input for DUKF. Moreover, after a simplification by the relationship between the TDPs, the parameter vector for TDPs UKF is set as:

(38) $$x_{p}={\left[m_{s}\;a\;b\right]}^{T}.$$

v is the observation noise vector, assumed to be zero mean Gaussian white noise with covariance R. There are $R=diag\left(\sigma_{{\ddot{z}}_{si}}^{2}\;\sigma_{{\dot{z}}_{si}}^{2}\;\sigma_{com_{i}}^{2}\right)$, and $\sigma_{{\ddot{z}}_{si}}^{}=0.01{\;m/s}^{2}$, $\sigma_{{\dot{z}}_{si}}^{}=0.01\;m/s$, $\sigma_{\Delta_{si}}^{}=0.1\;\mathrm{mm}$(i=fp,fd,rp,rd) [34]. The output vector is set as:

(39) $$y={\left[{\ddot{z}}_{sfd}{\ddot{z}}_{sfp}\;{\ddot{z}}_{srd}\;{\ddot{z}}_{srp}\;{\dot{z}}_{sfd}{\dot{z}}_{sfp}\;{\dot{z}}_{srd}\;{\dot{z}}_{srp}\;com_{fp}\;com_{fd}\;com_{rp}\;com_{rd}\right]}^{T}$$

where

(40) $$\left\{ \begin{array}{l} com_{fp}=\frac{m_{s}\times g\times \left(l_{x}-a\right)\times \left(l_{y}-b\right)}{\left(l_{x}\times l_{y}\right)\times k_{sfp}}+\Delta_{sfp}\\ com_{fd}=\frac{m_{s}\times g\times \left(l_{x}-a\right)\times b}{\left(l_{x}\times l_{y}\right)\times k_{sfd}}+\Delta_{sfd}\;\\ com_{rp}=\frac{m_{s}\times g\times a\times \left(l_{y}-b\right)}{\left(l_{x}\times l_{y}\right)\times k_{sfp}}+\Delta_{sfp}\;\\ com_{rd}=\frac{m_{s}\times g\times a\times b}{\left(l_{x}\times l_{y}\right)\times k_{sfp}}+\Delta_{sfp} \end{array}\right.\;$$

UT is a method for calculating the statistics of a random variable that undergoes a nonlinear transformation, which is detailed in Appendix B. The DUKF consists of the prediction parts and the update parts, and the formulas of the DUKF for such a nonlinear system are stated in Appendix C step-by-step.

## 5. Framework of Proposed Algorithm and Experimental Verification

**Figure 3:**
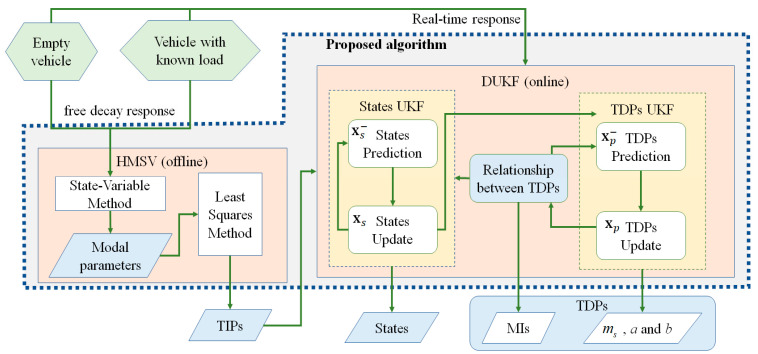
Flowchart of proposed algorithm.

A comprehensive algorithm is proposed to estimate TIPs as well as update TDPs and online states. A flowchart of the proposed algorithm is shown in Figure 3. TIPs are estimated by HMSV based on the free decay response of an empty vehicle and a vehicle with a known load. Substituting the estimated TIPs into the DUKF, both the TDPs and states can be derived online according to the real-time dynamic response of vehicle in practical driving. An experiment is conducted to estimate both TIPs and TDPs and the test data are used to validate the accuracy of the proposed algorithm.

A stage-drop experiment by a real vehicle was performed, and the proposed algorithm, illustrated in Figure 3, was applied to estimate the vehicle parameters. Considering the dropping in the test as a kind of driving condition, the real-time response can be used to identify TDPs for vehicles with or without loads. The additional mass could be easily measured and was used to verify the accuracy of the proposed algorithm.

**Figure 4:**
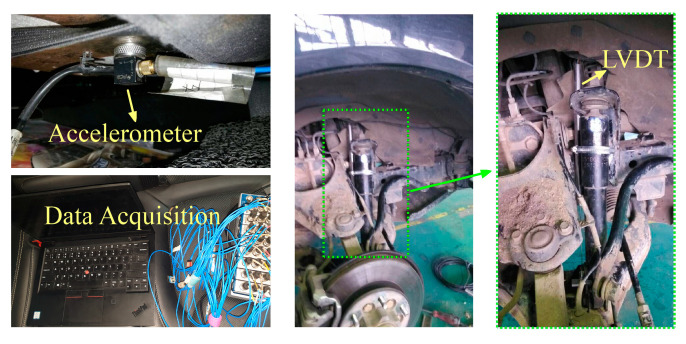
Tested vehicle and equipment.

An experiment was performed on a two-axle vehicle equipped with a National Instruments Data Acquisition, enabling the synchronization and the evaluation of the logged data. There are four accelerometers and four linear variable differential transformer (LVDT) sensors used to provide data in the tests. The accelerometers are PCB PIEZOTRONICS’s 356A17 model, providing accurate measurements of three-axis accelerations with a resolution of 0.1 mg. In this work, only the vertical acceleration is taken into consideration. Four accelerometers are mounted on the chassis near four springs. Four LVDT sensors are mounted at each suspension to collect the dynamic deflections. The resolution of LVDT is about 0.1 um. One each of the mounted accelerometers and LVDTs are shown in Figure 4.

The free-decay tests are dividing into two groups, with or without loading. Each group of tests includes a bounce test, pitch test and roll test of the vehicle. In the bounce test, four wheels of the vehicle are dropped simultaneously off stages with the same height. In the pitch test, the front or rear wheels are dropped off stages to carry out the pitch mode. Similarly, in the roll test, the left or right wheels are dropped off stages to carry out the roll mode.

The wheel distance $l_{y}$ and axle distance $l_{x}$ are measured directly and the CG of the empty vehicle can be determined by static compression measurement as:

(41) $$\left\{ \begin{array}{l} a_{0}=\frac{l_{x}\cdot com_{rp}}{com_{fp}+com_{rp}}\\ b_{0}=\frac{l_{y}\cdot com_{fd}}{com_{fp}+com_{fd}} \end{array}\right.$$

**Figure 5:**
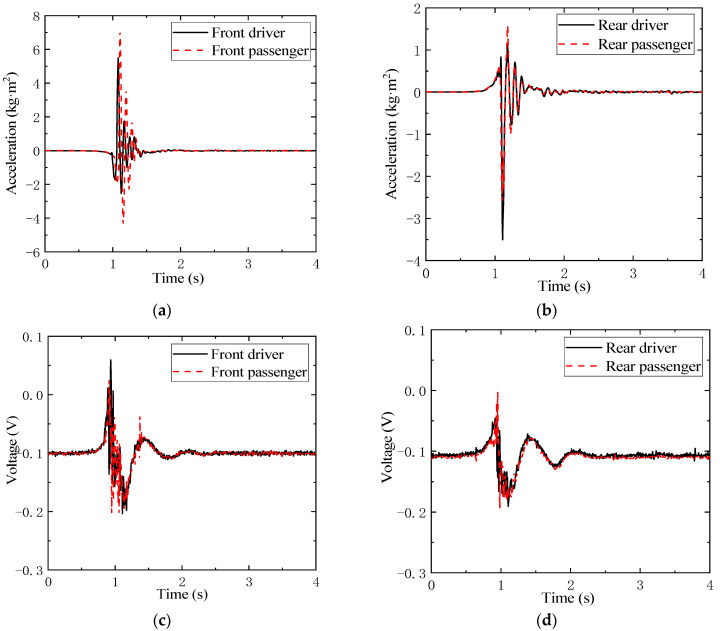
Measured data of accelerometer (**a**,**b**) and LVDT (**c**,**d**) in bounce test for empty vehicle.

These determinable distances are listed in Table 1. Furthermore, the read data of accelerometers and LVDTs in a bounce test for an empty vehicle are illustrated in the Figure 5. It can be seen from Figure 5a,b that the acceleration values at the same axle are similar, while for a different axle they are diverse, which indicates similar stiffness and damping for suspensions at the same axle, but different suspension parameters for the front and rear axles. The data collected by LVDT are voltages, which can be transferred to compressions of each suspension.

**Figure 6:**
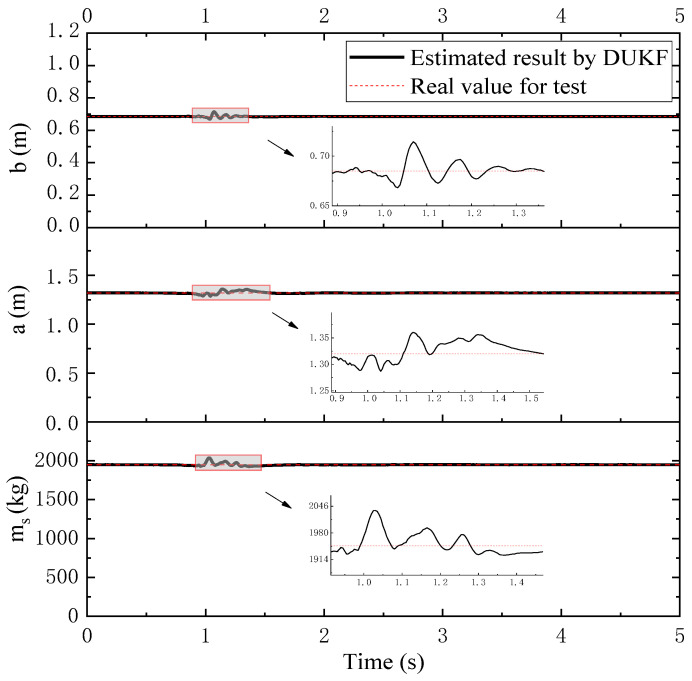
Estimated TDPs by experimental data.

Based on above test data, TIPs can be estimated by the HMSV part, as are listed in Table 2. With these estimated TIPs, TDPs can be further estimated by the DUKF part. The estimated $m_{s}$, a and b of the loading case are illustrated in Figure 6. The vehicle is in a steady state before 0.9 s and after 2 s, when a quite accurate mass and GC are obtained. It is worth noting that the dynamic deflections are nearly zero under a steady state, and Equation (40) gives the relationship between measured compressions and TDPs (mass and GC). In this way, TDPs can be estimated mainly according to the measured compression. This may be the reason why estimated TDPs keep nearly constant values for the steady state, considering that the data shown in Figure 5 are also smooth for the steady state. It can be seen from Figure 6 that the estimated values in the steady state are about 1948 kg, 1.32 mm and 0.68 mm, respectively. Furthermore, the additional mass Δm for the loading case is 390 kg, put at the CG of the empty vehicle. The CG of the loading case equals that of the empty case, and thus the values of a, b, $I_{x}$ and $I_{y}$ would equal to $a_{0}$, $b_{0}$, $I_{x0}$ and $I_{y0}$, respectively. Putting the estimated $m_{s}$, a and b into Equation (36), the estimated $I_{x}$ and $I_{y}$ can be determined as 600 kg·m^2^ and 2381 kg·m^2^, respectively. The estimated $m_{s}=m_{0}+\Delta m=1558+390=1948\;\mathrm{kg}$. All these indicate that the estimated parameters match the test results well. From 0.9 s to 1.5 s, the vehicle dropped and bounced, and at this time, both the accelerometer and LVDT data vibrated significantly, corresponding to Figure 5, which leads to a slight vibration of the estimated TDPs in Figure 6. The proposed algorithm can suppress the dynamic vibration of measured data because the state vector in Equation (36) includes the dynamic behavior of the vehicle. It can be seen from Figure 6 that the estimation errors for TDPs are small. A worse road condition may boost the estimation errors; the effect of road conditions on estimation accuracy will be further studied in the next section by a simulation. The states cannot be measured directly in the test; their estimation will be further verified with simulation results.

## 6. Performance Study of the Proposed Algorithm

In this section, further studies of the proposed algorithm are analyzed in terms of their ability to recognize the change of TDPs during a trip, the effect of nonlinear suspension properties and the performance on various road conditions, respectively. The simulations are implemented based on the vehicle model in Section 2. As TIPs will not change, these only need to be identified once and set as same for all cases. After each simulation, the estimation process is performed based on the set TIPs and the output vector in Equation (39). The accelerations and compressions of the output vector are calculated by adding Gaussian noise into the simulated sprung mass accelerations and suspension compressions, and the velocities of the output vector are obtained from the acceleration integral. TDPs and states change over time and are key to evaluate the algorithm’s performance.

### 6.1. Sprung Mass Variation

A simulation is performed for the shedding load at 4 s during the trip, with a driving speed 40 km/h on a rough road defined as road class B according to ISO 8608. Before shedding of the load happens, the load mass is 300 kg and corresponding $m_{s}=1858\;\mathrm{kg}$, a=1.62 m and b=0.885 m. After shedding part of the load, the load mass Δm is 150 kg and, correspondingly, $m_{s}=1708\;\mathrm{kg}$, a=1.47 m and b=0.785 m.

**Figure 7:**
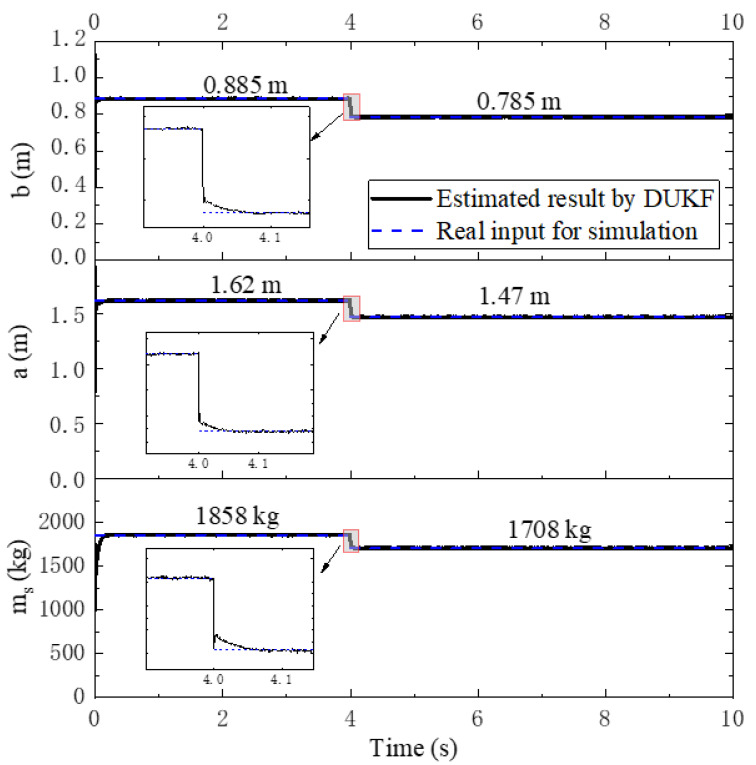
TDPs for simulation and estimation in the shedding load scenario.

Comparisons of the $m_{s}$, a and b values between the estimation and simulation are illustrated in Figure 7. Firstly, it can be seen that the estimated TDPs are nearly the same as the real input for the simulation if there is no change of loading, which means that the proposed algorithm is quite accurate for a class B road under 40 km/h. As the model used in the DUKF method accounts for the dynamic behavior of vehicle, the supposed algorithm can suppress the vibration. Secondly, at 4 s, shedding of the load happens and all TDPs change. It can be seen from Figure 7 that the proposed algorithm can detect the change of TDPs immediately, and approach the real input for simulation in about 0.05 s, which is quick enough for practical application.

**Figure 8:**
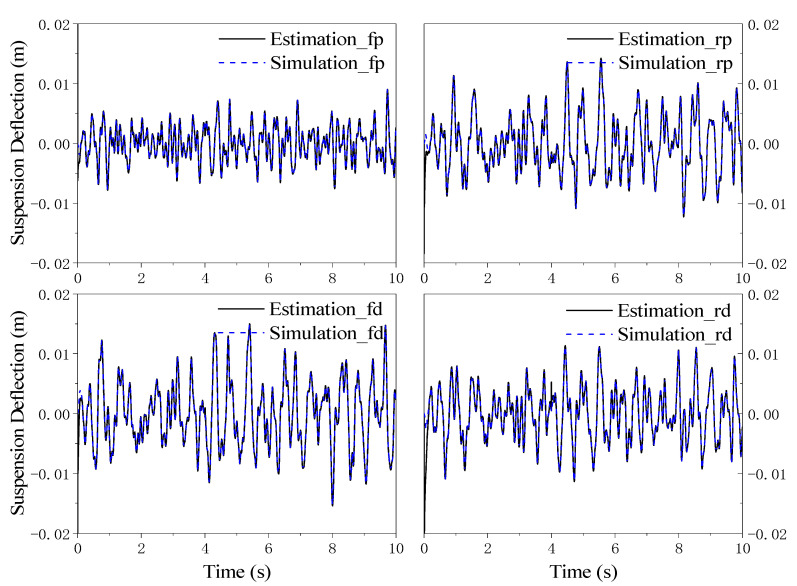
Suspension deflection for simulation and estimation in the shedding load scenario.

The dynamic deflections for the four suspensions can be estimated by DUKF, and their values in the mass change scenario are illustrated in Figure 8. It can be seen that the estimated values are nearly the same as the real inputs in the simulation, and even shedding a load at 4 s does not affect the estimated value by much. There is an approaching process happening near 0 s, which is because of the difference between the given initial values for DUKF and the real initial values in the simulation.

**Figure 9:**
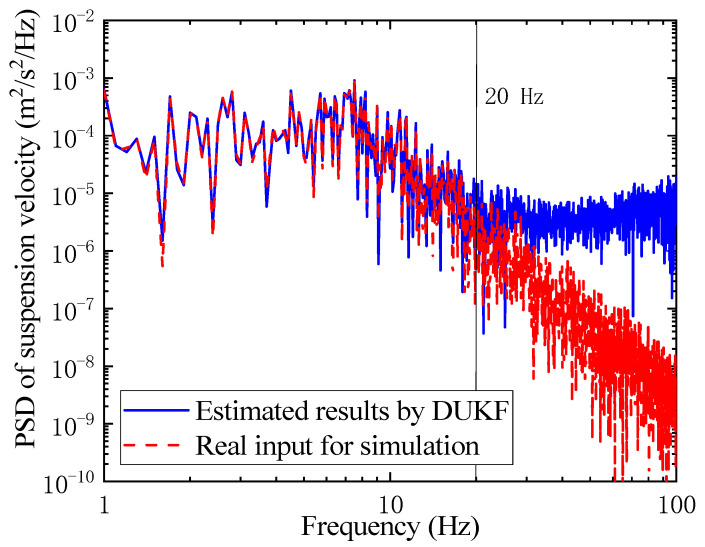
PSD of suspension velocity for simulation and estimation.

The suspension velocity can be estimated by the states UKF of the DUKF part in Figure 3. The power spectral density (PSD) values of the suspension velocity for the simulation and estimation are compared in Figure 9. It is clear that the estimated suspension velocity can approximate the real input for the simulation below 20 Hz, but in the higher frequency area, the estimated value is larger than the real input for the simulation.

**Figure 10:**
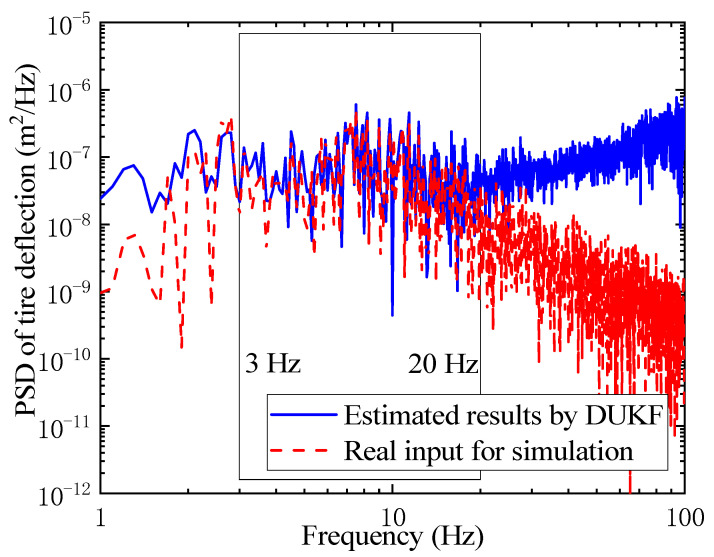
PSD of tire deflection for simulation and estimation.

Tire deflection can also be estimated by the DUKF part without road information. The PSDs of tire deflection for the simulation and estimation are shown in Figure 10, and it can be seen that the estimated tire deflection can approximate the real input for the simulation between 3 Hz and 20 Hz. The region of greatest human sensitivity to vertical vibration lies between 4 and 8 Hz [35], while the natural frequency of wheel-hop is about 12 Hz [36], both of which are in the relatively accurate region in Figure 10. Hence, the proposed algorithm can determine accurate tire deflection information in these frequency regions of concern.

### 6.2. Effect of Vehicle Model Linearization

The vehicle model given in Section 2 has linear stiffness and damping, but in reality, they are usually nonlinear [37]. Therefore, it is necessary to study the effect of linearization on the estimation results. The nonlinear part of the suspension reaction forces $f_{i}$ can be expressed as [38]:

(42) $$f_{i}=\left\{ \begin{array}{l} {k^{\prime }}_{si}\Delta_{si}{}^{3}+c_{1i}{\dot{\Delta }}_{si}\;\mathrm{if}\;\Delta_{si}\leq 0\;\\ {k^{\prime }}_{si}\Delta_{si}{}^{3}+c_{2i}{\dot{\Delta }}_{si}\;\mathrm{if}\;\Delta_{si}>0 \end{array}\right.(i=fp,fd,rp,rd),$$

where ${k^{\prime }}_{si}$ is the nonlinear part of suspension stiffness and $c_{1i}$ and $c_{2i}$ (i=fp,fd,rp,rd) are coefficients of suspension nonlinear damping.

In the simulations, the nonlinear parts of the suspension—${k^{\prime }}_{si}$, $c_{1i}$ and $c_{2i}$ (i=fp,fd,rp,rd)—are considered variables and the ratios of them to their corresponding linear parts are set from 0 percent to 50 percent, while for the estimation, only linear part values are taken into consideration. The relative error (*RE*) between the estimation and simulation values can be expressed as

(43) $$RE=\left|\frac{Simulation-Estimation}{Simulation}\right|.$$

**Figure 11:**
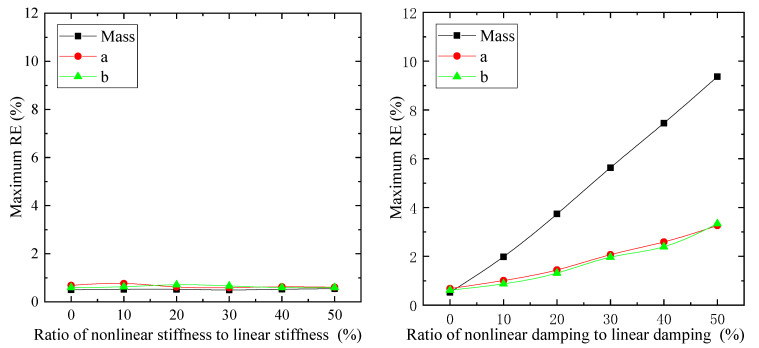
Maximum *RE* of TDPs for various ratios of nonlinear part to linear part for suspension properties.

The *RE* between the estimation and simulation represents the accuracy of the proposed estimation algorithm, and the maximum *RE*s of the TDPs are illustrated in Figure 11. It can be seen that various ratios of nonlinear stiffness to linear stiffness lead to a similar maximum *RE* (below 1%), whereas larger nonlinear damping causes higher *RE* values for TDPs. This means that disregard for nonlinear damping rather than nonlinear stiffness in the estimation process would affect the accuracy of TDPs. The reason could be that suspension deflections can be determined directly by the measured compressions, but the suspension velocity cannot. Accordingly, the incorrect stiffness value can be partly improved by the accurate suspension deflections with the help of the DUKF, whereas both the suspension velocity and the damping values are not accurate, which would lead to a large error eventually.

### 6.3. Performance under Various Road Conditions

**Figure 12:**
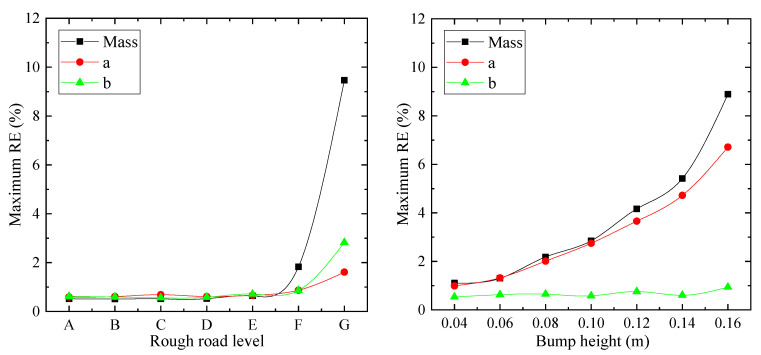
Maximum *RE* of TDPs for various ratios of nonlinear part to linear part for suspension properties.

In order to study the performance of the proposed estimation method under various road conditions, the simulations are implemented on various road roughness levels (according to ISO 8608) and speed bump heights. After that, the estimation process is performed based on the simulated results. A driving speed of 40 km/h and sprung mass of 2000 kg are considered for all cases. The *RE* values are calculated based on the simulation and estimation results, and the maximum *RE*s are illustrated in Figure 12. It can be seen that the proposed algorithm can maintain accurate estimation results with *RE*s of below 5% until road roughness level F. Even though the maximum *RE*s of $m_{s}$ reach about 10% for level G, in practice, it rarely happens that a vehicle is driving on level G under 40 km/h [39]. For the speed bump cases, the maximum *RE*s of $m_{s}$ and a increase with speed bump height. A commonly used height for speed bump is 0.07 m, on which the *RE* is below 2%. b does not change much; this is because vehicle is driven with two coaxial wheels travelling across the speed bump together. In total, the proposed algorithm can estimate TDPs with *RE*s of below 2% for road roughness level F and speed bump height 0.07 m, under 40 km/h.

### 6.4. Feasibility of the Algorithm for Updating TDPs

**Figure 13:**
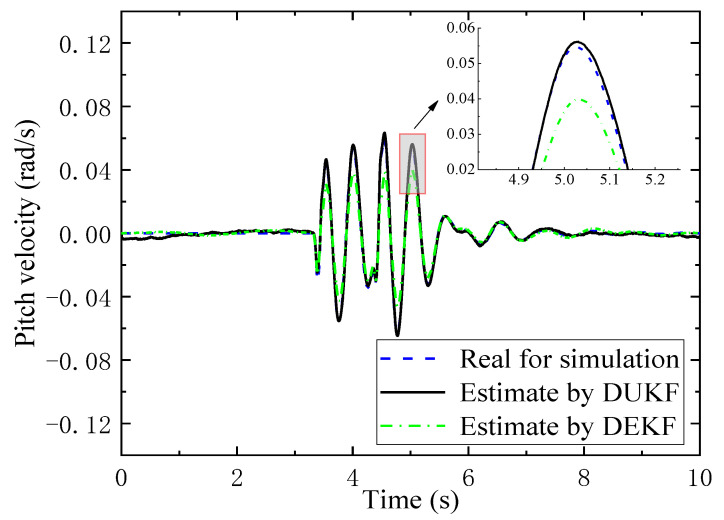
Comparison of the DUKF and DEKF estimating results.

The feasibility of the proposed algorithm from the implementation and computational point in practice is a very important question, and it is necessary to be studied. Therefore, we have illustrated a pitch velocity with real data in a simulation and estimated the results using the DUKF in Figure 13, where the results using the dual extended KF (DEKF) are compared to them. A detailed estimating process of the DEKF can be found in [40]. The simulation is conducted on a bumpy road (0.4 m height and 0.35 m width) under 10 km/h with a ratio of nonlinear stiffness force of 50% to the linear part. It can be seen from Figure 13 that the estimation errors by the DUKF are much smaller than that by the DEKF during the estimation process. The phenomenon can be explained by the DEKF using the linearization of Jacobian matrices with high-order truncation error to approximate the non-linear estimation system. Similar results for the comparison of accuracy between the DUKF and DEKF can also be found in [40].

For the 10 s simulation, the estimation of the DUKF uses 10.083 s to finish the estimation. It means the DUKF has a high possibility of updating TDPs online in practice. However, for the same simulation, the DEKF costs 2499.167 s. The most time-consuming process is the derivation of Jacobian matrices (2490.985 s) as there are fifteen variants in our state vector (Equation (36)), three variants in our parameter vector (Equation (38)) and twelve variants in our output vector (Equation (39)).

## 7. Conclusions

Identification of vehicle parameters is essential for dynamic analysis and control systems. This study is aimed to propose a fusion algorithm for identifying comprehensive vehicle parameters without the help of an inertial parameter measurement device (IPMD). This paper divides the parameters of the vehicle into time-independent parameters (TIPs) and time-dependent parameters (TDPs) based on whether or not they change over time. The novelty of this paper is providing a comprehensive algorithm to estimate TIPs as well as update TDPs and online states for vehicle. TIPs were identified firstly by means of a hybrid-mass state-variable (HMSV) method. With the estimated values of TIPs, a dual unscented Kalman filter (DUKF), consisting of two parallel layers running simultaneously, was constructed to estimate both TDPs and online states. Experiments were conducted to estimate both TIPs and TDPs and so as to validate the accuracy of proposed algorithm. Numerical simulations were performed to further investigate the DUKF’s performance in terms of sprung mass variation, model error during linearizing and various road conditions. The results from both the experiment and the simulation showed that the proposed algorithm could estimate TIPs as well as update TDPs and online states with high accuracy and no requirement of the road information.

With the proposed method, vehicle parameters can be identified separately and accurately. TIPs can be identified offline and stored for further use, while only TDPs are updated online, which improves the efficiency of estimation. In addition, it worth noting that by including the relationship between dynamic states and compression, the accuracy of the proposed method is quite high except for under significant dynamics caused by very bad road conditions, such as Class G roads or high bumps. We are now interested in extending the method for other mechanical systems in the future.

## Figures and Tables

**Table 1:** Determinable distances and CG of empty vehicle.

Parameters	Values	Units
$l_{x}$	1.37	m
$l_{y}$	2.7	m
$a_{0}$	1.32	m
$b_{0}$	0.685	m

**Table 2:** Estimation results of TIPs with experimental data.

Parameters	Values	Units
$m_{0}$	1558	kg
$I_{y0}$	2381	kg·m^2^
$I_{x0}$	600	kg·m^2^
$m_{ufd}$ $m_{ufp}$	96.4	kg
$m_{urd}$ $m_{urp}$	105.6	kg
$k_{sfd}$ $k_{sfp}$	36,557	N/m
$c_{sfd}$ $c_{sfp}$	3200	N·s/m
$k_{srd}$ $k_{srp}$	42,677	N/m
$c_{srd}$ $c_{srp}$	3500	N·s/m
$k_{ti}$ *(* i=fp,fd,rp,rd *)*	200,900	N/m

## Nomenclature

**Notation**	**Description**
z	Vertical Displacement
θ	Pitch Motion
ϕ	Roll Motion
$\ddot{X}$	Acceleration Vector
$\dot{X}$	Velocity Vector
X	Displacement Vector
M	Mass Matrix
C	Damping Matrix
K	Stiffness Matrix
F	Force Matrix
Y	State Vector
A and B	Coefficient Matrix
Π and Γ	State Matrices
dt	Sampling Interval
R	Measure Error Matrix
λ	Eigenvalue
U	Eigenvector
**Subscript**	
s	Sprung Mass
u	Unsprung Mass
f	Front Axle
r	Real Axle
d	Driver Side
p	Passenger Side
ext	External Excitation
non	Nonlinear Part
new	New Mass
**Superscript**	
sub	Submatrices
∗	Conjugate Matrices

## Appendix A. Vehicle Parameters

**Table A1:** Fixed parameters for vehicle.

Parameter	Description	Unit
$m_{0}$	Empty sprung mass	kg
$I_{x0}$	Roll MI of empty sprung mass	$\mathrm{kg}\cdot m^{2}$
$I_{y0}$	Pitch MI of empty sprung mass	$\mathrm{kg}\cdot m^{2}$
$m_{ui}$(i=fp,fd,rp,rd)	Unsprung mass	kg
$k_{si}$(i=fp,fd,rp,rd)	Stiffness of suspension spring	N/m
$k_{ti}$(i=fp,fd,rp,rd)	Stiffness of tire	N/m
$c_{si}$(i=fp,fd,rp,rd)	Damping of suspension	N⋅s/m
$l_{y}$	Axle distance	m
$l_{x}$	Wheel distance at same axle	m
$a_{0}$	Distance from CG to front axle for empty vehicle	m
$b_{0}$	Distance from CG to passenger side wheel line for empty vehicle	m

**Table A2:** Time-dependent parameters for vehicle.

Parameter	Description	Unit
$m_{s}$	Total sprung mass	kg
Δm	Additional sprung mass	kg
$I_{x}$	Roll MI of real-time sprung mass	$\mathrm{kg}\cdot m^{2}$
$I_{y}$	Pitch MI of real-time sprung mass	$\mathrm{kg}\cdot m^{2}$
a	Distance from real-time CG to front axle	m
b	Distance from real-time CG to passenger side wheel line	m

## Appendix B. Unscented Transform

The unscented transform (UT) is used to deal with the nonlinear part. Using state UT as an example, the 2N + 1 sigma points are chosen as:

(A1) $$x_{s}^{}{}^{\left(i\right)}=UT\left({\bar{x}}_{s}^{},\;P_{s}^{}\right)=\left\{ \begin{array}{ll} {\bar{x}}_{s}^{},\; & i=0\;\\ {\bar{x}}_{s}^{}+{\left(\sqrt{\left(N+\lambda \right)P_{s}^{}}\right)}^{\left(i\right)},\; & i=1,\ldots ,N\\ {\bar{x}}_{s}^{}-{\left(\sqrt{\left(N+\lambda \right)P_{s}^{}}\right)}^{\left(i-N\right)},\; & i=N+1,\ldots ,2N \end{array}\right.$$

*N* is the dimension of the state vector $x_{s}$; $\lambda =\varepsilon_{1}{}^{2}\left(N+\kappa \right)-N$ is a scaling parameter, user-defined [41]; the constant $\varepsilon_{1}$ is usually set as $\left[1\times 10^{-4},1\right]$, determining the spread of the sigma around the mean of these sigma points ${\bar{x}}_{s}^{}$; κ≥0 can make sure that the covariance matrix is definitely positive. The weights of the sigma points are calculated as:

(A2) $$\left\{ \begin{array}{ll} w^{m}{}^{\left(0\right)}=\frac{\lambda }{N+\lambda },\; & i=0\\ w^{c}{}^{\left(0\right)}=\frac{\lambda }{N+\lambda }+1-\varepsilon_{1}{}^{2}+\varepsilon_{2},\; & i=1,\ldots ,2N\;\\ w^{m}{}^{\left(i\right)}=w^{c}{}^{\left(i\right)}=\frac{1}{2\left(N+\lambda \right)},\; & i=1,\ldots ,2N\; \end{array}\right.$$

$\varepsilon_{2}$ considers the high-order moment of the prior distribution, and it is optimally set as 2 for Gaussian distribution.

## Appendix C. Formulas of the DUKF

The DUKF consists of the prediction parts and the update parts, and the formulas of the DUKF for such a nonlinear system are stated here step-by-step:

Step 1. State prediction

(A3) $$x_{s,k-1}{}^{\left(i\right)}=UT\left(x_{s,k-1},P_{s,k-1}\right)$$

(A4) $$x_{s,k}^{*}{}^{\left(i\right)}=f\left(x_{s,k-1}{}^{\left(i\right)},x_{p,k-1}^{},u_{k}\right)$$

(A5) $$x_{s,k}^{-}=\sum_{i=0}^{2N_{s}}\left(w^{m\left(i\right)}\cdot x_{s,k}^{*}{}^{\left(i\right)}\right)$$

(A6) $$P_{s,k}^{-}=\sum_{i=0}^{2N_{s}}\left(w^{c\left(i\right)}\cdot \left(x_{s,k}^{*}{}^{\left(i\right)}-x_{s,k}^{-}\right){\left(x_{s,k}^{*}{}^{\left(i\right)}-x_{s,k}^{-}\right)}^{T}\right)+Q_{s,k-1}$$

Step 2. State measure update

(A7) $$x_{s,k}^{-}{}^{\left(i\right)}=UT\left(x_{s,k}^{-},P_{s,k}^{-}\right)$$

(A8) $$y_{k}^{*}{}^{\left(i\right)}=h\left(x_{s,k}^{-}{}^{\left(i\right)},x_{p,k-1}^{},u_{k}\right)$$

(A9) $${\bar{y}}_{k}^{}=\sum_{i=0}^{2N_{s}}\left(w^{m\left(i\right)}\cdot y_{k}^{*}{}^{\left(i\right)}\right)$$

(A10) $$P_{y_{k}y_{k}}^{}=\sum_{i=0}^{2N_{s}}\left(w^{c\left(i\right)}\cdot \left(y_{k}^{*}{}^{\left(i\right)}-{\bar{y}}_{k}^{}\right){\left(y_{k}^{*}{}^{\left(i\right)}-{\bar{y}}_{k}^{}\right)}^{T}\right)+R$$

(A11) $$P_{x_{k}y_{k}}^{}=\sum_{i=0}^{2N_{s}}\left(w^{c\left(i\right)}\cdot \left(x_{s,k}^{-}{}^{\left(i\right)}-x_{s,k}^{-}\right){\left(y_{k}^{*}{}^{\left(i\right)}-{\bar{y}}_{k}^{}\right)}^{T}\right)$$

Step 3. State update

(A12) $$K_{s,k}=P_{x_{k}y_{k}}^{}P_{y_{k}y_{k}}^{}{}^{-1}$$

(A13) $$x_{s,k}=x_{s,k}^{-}+K_{s,k}\left(y_{k}-{\bar{y}}_{k}^{}\right)$$

(A14) $$P_{s,k}^{}=P_{s,k}^{-}-K_{s,k}P_{y_{k}y_{k}}^{}K_{s,k}{}^{T}$$

Step 4. SIP prediction

(A15) $$x_{p,k}^{-}=x_{p,k-1}^{}$$

(A16) $$p_{p,k}^{-}=p_{p,k-1}^{}+Q_{p}$$

Step 5. SIP measure update

(A17) $$x_{p,k}^{-}{}^{\left(i\right)}=UT\left(x_{p,k}^{-},p_{p,k}^{-}\right)$$

(A18) $$x_{s(p),k}^{*}{}^{\left(i\right)}=f\left(x_{s,k-1},x_{p,k}^{-}{}^{\left(i\right)},u_{k}\right)$$

(A19) $$y_{p,k}^{*}{}^{\left(i\right)}=h\left(x_{s(p),k}^{*}{}^{\left(i\right)},x_{p,k}^{-}{}^{\left(i\right)},u_{k}\right)$$

(A20) $${\bar{y}}_{p,k}^{}=\sum_{i=0}^{2N_{p}}\left(w^{m_{p}\left(i\right)}\cdot y_{p,k}^{*}{}^{\left(i\right)}\right)$$

(A21) $$P_{y_{pk}y_{pk}}^{}=\sum_{i=0}^{2N_{p}}\left(w^{c_{p}\left(i\right)}\cdot \left(y_{p,k}^{*}{}^{\left(i\right)}-{\bar{y}}_{p,k}^{}\right){\left(y_{p,k}^{*}{}^{\left(i\right)}-{\bar{y}}_{p,k}^{}\right)}^{T}\right)+R$$

(A22) $$P_{x_{k}y_{pk}}^{}=\sum_{i=0}^{2N_{p}}\left(w^{c_{p}\left(i\right)}\cdot \left(x_{p,k}^{-}{}^{\left(i\right)}-x_{p,k}^{-}\right){\left(y_{p,k}^{*}{}^{\left(i\right)}-{\bar{y}}_{p,k}^{}\right)}^{T}\right)$$

Step 6. SIP update

(A23) $$K_{p,k}=P_{x_{k}y_{pk}}^{}P_{y_{pk}y_{pk}}^{}{}^{-1}$$

(A24) $$x_{p,k}=x_{p,k}^{-}+K_{p,k}\left(y_{k}-{\bar{y}}_{p,k}\right)$$

(A25) $$P_{p,k}^{}=P_{p,k}^{-}-K_{p,k}P_{y_{pk}y_{pk}}^{}K_{p,k}{}^{T}$$

where $N_{s}$ and $N_{p}$ are the dimensions of the state vector and parameter vector, respectively. $y_{k}$ are measurement data.
